# The Omega-3 Index and Self-Reported Dietary Intake of DHA and EPA in Highly Trained, Elite and World Class (Tier 3–5) Male and Female Athletes: An Australian Based Cross-sectional Study

**DOI:** 10.1186/s40798-026-01070-5

**Published:** 2026-07-17

**Authors:** Michael J. Macartney, Ryan Anthony, Joel Craddock, Marijka Batterham, Gregory E. Peoples

**Affiliations:** 1 School of Medical Indigenous & Health Sciences, Faculty of Science Medicine and Health, University of Wollongong, Wollongong, NSW 2525 Australia; 2 School of Clinical Medicine, Rural Clinical Campuses, University of New South Wales, Sydney, NSW Australia; 3 School of Health Sciences, University of New South Wales, Sydney, NSW Australia; 4 Graduate School of Medicine, University of Wollongong, Wollongong, NSW Australia

**Keywords:** Omega-3 Index, DHA and EPA intake, Athletes, Sports nutrition, Cardiovascular disease risk

## Abstract

**Background:**

Long-chain omega-3 fatty acids docosahexaenoic acid (DHA) and eicosapentaenoic acid (EPA) support cardiovascular health; however, athletes often exhibit Omega-3 Index (O3I) levels below 8%, a threshold associated with reduced cardiovascular disease (CVD) risk, suggesting insufficient dietary intake of DHA and EPA. Therefore, the objective of this study was to characterise the O3I, dietary intake of DHA and EPA, and supplement use in a large cohort of highly trained, elite and world class (Tier 3–5) Australian based athletes.

**Methods:**

In this multi-site cross-sectional study, 595 male and female athletes (18–45 years) from 17 sports provided a dried blood spot for whole blood fatty acid analysis and completed a dietary survey. The O3I was derived using a validated conversion algorithm. Dietary DHA and EPA intake was assessed using a marine food frequency questionnaire. Group comparisons were conducted using Mann–Whitney U and independent-samples t-tests, and differences across intake quartiles were assessed using Kruskal–Wallis tests.

**Results:**

Mean (SD) O3I was 5.88 ± 1.40%, with 3% of athletes < 4%, 60% between 4–6%, 30% between 6–8%, and only 7% ≥ 8%. O3I increased across DHA + EPA intake quartiles (p < 0.001) and was higher in males than females (p < 0.01). Athletes reporting omega-3 supplement use (19%) had higher O3I (Median [IQR]) compared with non-users (7.02 [6.28–8.01%] vs 5.38 [4.82–6.02%]; p < 0.001). Median total daily DHA + EPA intake was 261 mg/day, with 72% of athletes consuming < 500 mg/day. Daily whole-food intake of DHA + EPA was modest and did not differ by sex.

**Conclusion:**

Most athletes based in Australia had O3I below the ≥ 8% CVD risk reduction threshold, highlighting opportunities for organisations and sporting bodies to support initiatives that increase DHA and EPA intake.

## Key Points

- The mean Omega-3 Index (O3I) in highly trained, elite and world class (Tier 3–5) Australian based athletes was 5.88%. Despite representing the highest values reported in an athlete cohort, only ~ 7% of athletes achieved the cardiovascular disease risk reduction threshold of ≥ 8%.
- Higher self-reported docosahexaenoic acid (DHA) and eicosapentaenoic acid (EPA) intake from whole foods and supplement use achieved a higher O3I; however, aside from a very small number of individuals, only those in the highest self-reported intake quartile (> 550 mg/day) approached or exceeded 8%.
- Dietary DHA and EPA intake is a modifiable determinant of O3I in athletes and may be addressed through individual or team-based strategies, with O3I serving as a biomarker associated with cardiovascular health across the athlete lifespan.

## Introduction

Long-chain omega-3 polyunsaturated fatty acids (LCn-3PUFA), specifically docosahexaenoic acid (DHA) and eicosapentaenoic acid (EPA), are incorporated into cell membranes and contribute to the structural and functional integrity of cardiac, skeletal muscle and neural tissues [1–3]. At a population level, higher DHA and EPA intake is associated with reduced risk for cardiovascular, neurodegenerative and inflammatory conditions [4]. From an exercise perspective, modulation of whole-body and neural inflammation [5, 6], enhanced skeletal muscle recovery [7] and modification of heart rate [8] underscore the potential of DHA and EPA to provide nutritional “pre-conditioning” to support the demands of training and competition. Given these emerging themes, there is increasing interest in the role of DHA and EPA in physically trained populations [9], including whether athletes achieve sufficient dietary intake for tissue incorporation and how this relates to dose, duration, and physiological outcomes.

The Omega-3 Index (O3I), defined as the proportion of DHA and EPA in erythrocyte membranes, provides an objective biomarker of long-term dietary omega-3 intake. An O3I ≥ 8% is widely considered indicative of desirable levels based on cardiovascular disease risk outcomes in general population [10], and reflects sustained dietary intake of DHA and EPA; however, its applicability as an athlete-specific target remains to be established. Despite this, early observations in German elite winter sport athletes, undertaken on the basis of O3I as a cardiovascular disease risk marker, reported values < 5% [11]. Since then, evidence from physically trained cohorts consistently demonstrates O3I values below this threshold, with mean values typically reported in the range of 4 to 6% across athletes from a range of sports [11–18]. This is notable given that endurance athletes have also been shown to develop distinct cardiac adaptations, including an increased prevalence of coronary artery plaques compared to sedentary adults [19, 20]. Within the Australian context, these O3I findings have been preliminarily confirmed. In elite rugby football athletes, mean O3I values of 5–6% were reported, with only a small proportion of individuals achieving the proposed target of ≥ 8% [21, 22]. Similar observations have been made in endurance athletes, where baseline O3I values reflect habitual dietary patterns of insufficient preformed DHA and EPA intake [23] exemplified by endurance athletes following a plant based diet [24] and aligning with O3I typically observed in the general population [25].

While increasing dietary DHA and EPA intake is an established method for elevating the O3I, a clear gap remains between theoretical recommendations and real-world practice [21]. Dietary achievable intakes of ~ 750–1500 mg/day DHA + EPA can meaningfully increase O3I, with ~ 840 mg/day elevating O3I from ~ 4.9% to ~ 6.5% within ~ 13 weeks [26]. Interestingly, higher O3I levels observed with increasing age, even after adjustment for dietary intake, suggest that longer-term exposure or age-related factors may contribute to achieving and maintaining elevated O3I [27]. As a result, O3I ≤ 6% appears to be a consistent feature of athletic populations, including those competing at the highest levels. However, current evidence is largely derived from single-sport or small cohort studies conducted within specific performance environments, limiting the ability to understand broader patterns of O3I and intake of DHA and EPA across the high-performance sport system. This is particularly relevant in Australia, where dietary behaviours and access to nutrition support may vary across sporting codes and organisational contexts.

Therefore, the aim of the present study was to characterise dietary intake of marine-derived DHA and EPA, omega-3 supplement use, and whole blood fatty acid levels in a large cohort of highly trained, elite and world class (Tier 3–5) athletes [28], based in Australia. The primary outcome was the O3I, with secondary outcomes including whole blood fatty acid profiles, dietary intake patterns, supplement use and exploring sex-based differences.

## Methods

### Study Design and Participants

This cross-sectional study was conducted as a community embedded and multi-site design (nation-wide) between March 2023 and December 2025. Male and female volunteers (18–45 years of age), who were training and competing as either individuals or in team sports, were recruited to provide a finger prick blood sample and complete a short survey regarding their demographics and diet. Recruitment took place using direct approach with high performance or nutrition/dietitian staff associated with either the athlete or their squads and teams. In addition, a snowball method was employed whereby further participants were included through recommendation. The participants level of athletic attainment included representation at either Tier 3 (highly trained/national level), Tier 4 (elite/international level) or Tier 5 (world-class competition) [28]. Exclusion criteria included current illness or injury, or inability to participate in regular training and competition for the preceding 6 months due to illness or injury. Ethics approval was obtained from the University of Wollongong Human Research Ethics Committee (REF no. H2025-0844), and all participants provided informed consent prior to participation.

### Whole Blood Fatty Acids and Omega-3 Index

Participants were requested to provide a blood sample (non-fasted) using the finger prick method to obtain a dry blood spot. They were instructed not to consume any form of omega-3 supplement on the day of blood collection. The fingertip was first cleaned using an alcohol swab and a small lancet was then used to puncture the skin. Enough blood was spotted onto the commercially available collection card, containing antioxidant, to fill a predefined area and allowed to dry. The samples were then sent for independent analysis at a commercially available laboratory (Fatty Acid Labs, Melbourne, Australia). Gas chromatography methods were used to separate each fatty acid and individually identified them using high quality standards. Each fatty acid was then described as a relative percentage (%) of all the fatty acids. The O3I was calculated from the whole blood DHA and EPA values using a validated conversion algorithm to derive erythrocyte-equivalent %DHA + EPA values (r = 0.96) [29].

### Demographics and Dietary Survey

Participants were requested to independently complete a short survey using their personal devices. These survey questions were sent directly to the participant using REDCap electronic data capture tools [30], hosted at the University of Wollongong, to limit influence from performance team expectations. In the first section of the survey, participants were asked to report their sex, age, height and body mass. They were also required to complete details regarding their primary sport of training and competition. The second section of the survey was then focussed on a marine food frequency and supplementation questionnaire pertaining to the month preceding blood sampling. The questions were derived from a previously validated Australian survey [31], and consisted of two components which sought to capture usual marine DHA and EPA intake from whole foods using two items; (i) portion size of fish reported using six predefined categories (85–227 g), and (ii) consumption frequency over the previous month was recorded across seven marine food groups: (1) oily fish (fresh or canned salmon, herring, mackerel, sardines); (2) canned tuna; (3) trout or halibut; (4) white fish (sole, rockfish, haddock, cod, etc.); (5) molluscs (mussels, oysters, clams, scallops); (6) crustaceans (shrimps, crabs, lobsters, etc.); and (7) imitation crab, using eight categories: (1) Never; (2) Once a month; (3) 2–3 times per month; (4) Once a week; (5) 2–3 times per week; (6) 4–6 times per week; (7) Once a day; (8) I don't know/refuse to answer.

Daily whole food intakes (g/day) were calculated from portion size and frequency, and corresponding EPA and DHA intakes (mg/day) were derived using food-specific fatty acid values. Australian NUTTAB 2010 data were used to assign the total DHA and EPA content to each food group, acknowledging inherent variability and limitations of food composition databases. Additional questions relating to DHA and EPA supplementation were modified to capture supplement brand, duration of supplementation (months), frequency of consumption (days per week) and quantity (capsules or volume) of supplements consumed. From these questions, a total intake of DHA and EPA (mg/day) from (i) whole food sources, (ii) supplementation and (iii) combined whole food and supplementation was determined for each participant.

### Statistical Analysis

Statistical analyses were performed using IBM SPSS (version 30, IBM, Armonk NY). Data distributions were assessed using histograms and measures of skewness and kurtosis. Due to non-normal distributions, continuous variables are presented as median ± interquartile range (IQR) with ranges, unless otherwise stated. Variables meeting assumptions of normality, including whole blood fatty acid proportions and O3I, are presented as mean ± standard deviation (SD). Survey responses of ‘I don’t know/refuse to answer’ were treated as missing and excluded from analyses. Group comparisons (males vs. females; supplement users vs non-users) were conducted using Mann–Whitney U tests for non-normally distributed variables and independent-samples t-tests for normally distributed variables. The O3I was compared across quartiles of self-reported total daily DHA and EPA intake using Kruskal–Wallis tests. Statistical significance was set at p < 0.05. Figures were generated using GraphPad Prism (version 10.6.1; GraphPad Software, San Diego, CA, USA).

## Results

### Participant Characteristics

A total of five hundred and ninety-five athletes (n = 595) from 17 sporting codes were represented in the cohort. Australian football (14%), rugby union (13%), rugby league (12%) and soccer (10%) comprised the largest proportions of athletes. Athletics (9%), cricket (8%), alpine sports (7%) and hockey (6%) were also represented. Smaller proportions of athletes competed in basketball (4%), canoe slalom (3%), powerlifting (3%), water polo (3%), swimming (2%), golf (2%), netball (2%), cycling (1%) and mixed martial arts (1%). Sports were classified as contact or non-contact based on the presence or absence of routine bodily collision during competition. Athletes represented samples from the following Australian states: New South Wales, Victoria, Queensland, South Australia and Tasmania. The cohort had slightly more males and most athletes competed in contact, team-based sports (Table 1). Whole blood samples were provided by all athletes, while dietary intake data were available for a subset of athletes.

**Table 1:** Demographic characteristics of athletes

	Whole cohort	Male	Female
Whole blood sample	595 (100%)	341 (57%)	254 (43%)
Self-report dietary intake	460	245	215
Training and Performance Caliber*			
Tier 3 (highly trained/national level)	117	53	64
Tier 4 (elite/international level)	334	198	136
Tier 5 (world-class competition)	144	90	54
Age (years)	26 ± 5	27 ± 5	25 ± 5
Height (m)	1.79 ± 0.11	1.85 ± 0.09	1.71 ± 0.07
Body mass (kg)	80.4 ± 15.8	87.5 ± 14.3	70.7 ± 12.1
BMI (kg/m^2^)	24.9 ± 3.5	25.5 ± 3.4	24.3 ± 3.5
Sport type			
Contact sport	322	190	132
Non-contact sport	273	151	122
Sport structure			
Team sport	433	241	192
Individual sport	162	100	62

Values are presented as n (%), n or mean ± SD. All athletes completed dry blood spot. Dietary intake assessment data were available for a subset of athletes that agreed to complete it. * Participants’ training and performance level were classified according to the framework described by McKay et al. [28]

### Omega-3 Index and Whole Blood Fatty Acids

Trans fatty acids accounted for a small proportion of total whole blood fatty acids, with males exhibiting slightly lower levels than females (Table 2). Saturated fatty acids comprised the largest relative proportion of the whole blood fatty acid profile. Total saturated fatty acid levels did not differ by sex; however, males exhibited higher palmitic acid, while stearic acid was marginally lower in females. Monounsaturated fatty acids accounted for approximately one quarter of the total whole blood fatty acid profile, with no sex differences observed. Total omega-6 whole blood fatty acid levels were similar between sexes, although females exhibited higher linoleic acid, whereas arachidonic acid was modestly higher in males. Total omega-3 whole blood fatty acids were higher in males than females, driven by higher EPA, DPA, and DHA levels, while alpha-linolenic acid contributed minimally to the total omega-3 levels.

**Table 2:** Whole blood fatty acids collected from highly trained, elite and world class (Tier 3–5) male and female Australian based athletes

	Whole cohort	Male	Female	p-value
Σ Trans fatty acids	0.70 ± 0.24 (0.13–2.16)	0.68 ± 0.21	0.74 ± 0.26	0.003
Σ Saturated fatty acids	35.75 ± 1.61 (27.21–41.87)	35.68 ± 1.62	35.86 ± 1.60	0.180
Palmitic acid (*16:0*)	21.52 ± 1.28 (17.04–26.93)	21.37 ± 1.18	21.73 ± 1.37	< 0.001
Stearic acid (*18:0*)	12.10 ± 0.96 (8.14–15.24)	12.20 ± 0.94	11.97 ± 0.97	0.003
Σ Monounsaturated fatty acids	22.11 ± 2.39 (16.24–35.22)	22.11 ± 2.43	22.11 ± 2.35	0.999
Oleic acid (*18:1n9*)	20.52 ± 2.30 (15.18–33.79)	20.55 ± 2.33	20.48 ± 2.25	0.736
Σ Omega-6 fatty acids	35.22 ± 2.45 (23.96–40.18)	35.09 ± 2.56	35.39 ± 2.29	0.148
Linoleic acid (LA, *18:2n6*)	21.41 ± 2.37 (14.42–29.25)	21.20 ± 2.35	21.70 ± 2.36	0.011
Arachidonic acid (AA, *20:4n6*)	10.09 ± 1.50 (4.13–15.18)	10.22 ± 1.48	9.92 ± 1.52	0.014
Σ Omega-3 fatty acids	6.22 ± 1.36 (3.27–12.01)	6.44 ± 1.41	5.91 ± 1.24	< 0.001
Alpha-Linolenic acid (ALA, *18:3n3*)	0.60 ± 0.24 (0.20–2.27)	0.57 ± 0.22	0.64 ± 0.26	< 0.001
Eicosapentaenoic acid (EPA, *20:5n3*)	0.98 ± 0.60 (0.16–4.85)	1.05 ± 0.62	0.89 ± 0.54	< 0.001
Docosapentaenoic acid (DPA, *22:5n3*)	1.61 ± 0.39 (0.72–3.74)	1.71 ± 0.41	1.48 ± 0.32	< 0.001
Docosahexaenoic acid (DHA, *22:6n3*)	3.02 ± 0.78 (1.30–5.99)	3.11 ± 0.79	2.90 ± 0.76	0.002

Values for the whole cohort are mean ± SD (min–max). Values for male and female are mean ± SD. Between-sex comparisons were conducted using independent-samples t-tests or Mann–Whitney tests for non-normal data. p-values were derived using Welch’s correction where variances were unequal

**Fig. 1:**
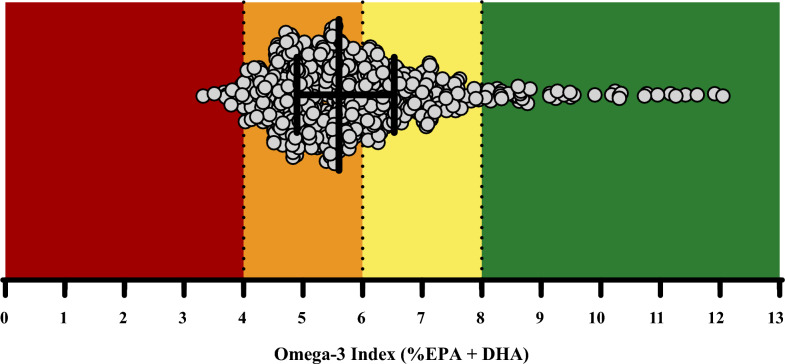
Distribution of O3I values in a cohort of highly trained, elite and world class (Tier 3–5) Australian-based male and female athletes. Individual observations are shown as open circles. The central bar represents the median, with outer bars indicating the interquartile range. Shaded regions denote commonly used O3I risk thresholds (red: < 4%, highest risk; orange/yellow: 4–8%, intermediate risk; green: ≥ 8%, lowest risk) as defined by Harris [10]

**Fig. 2:**
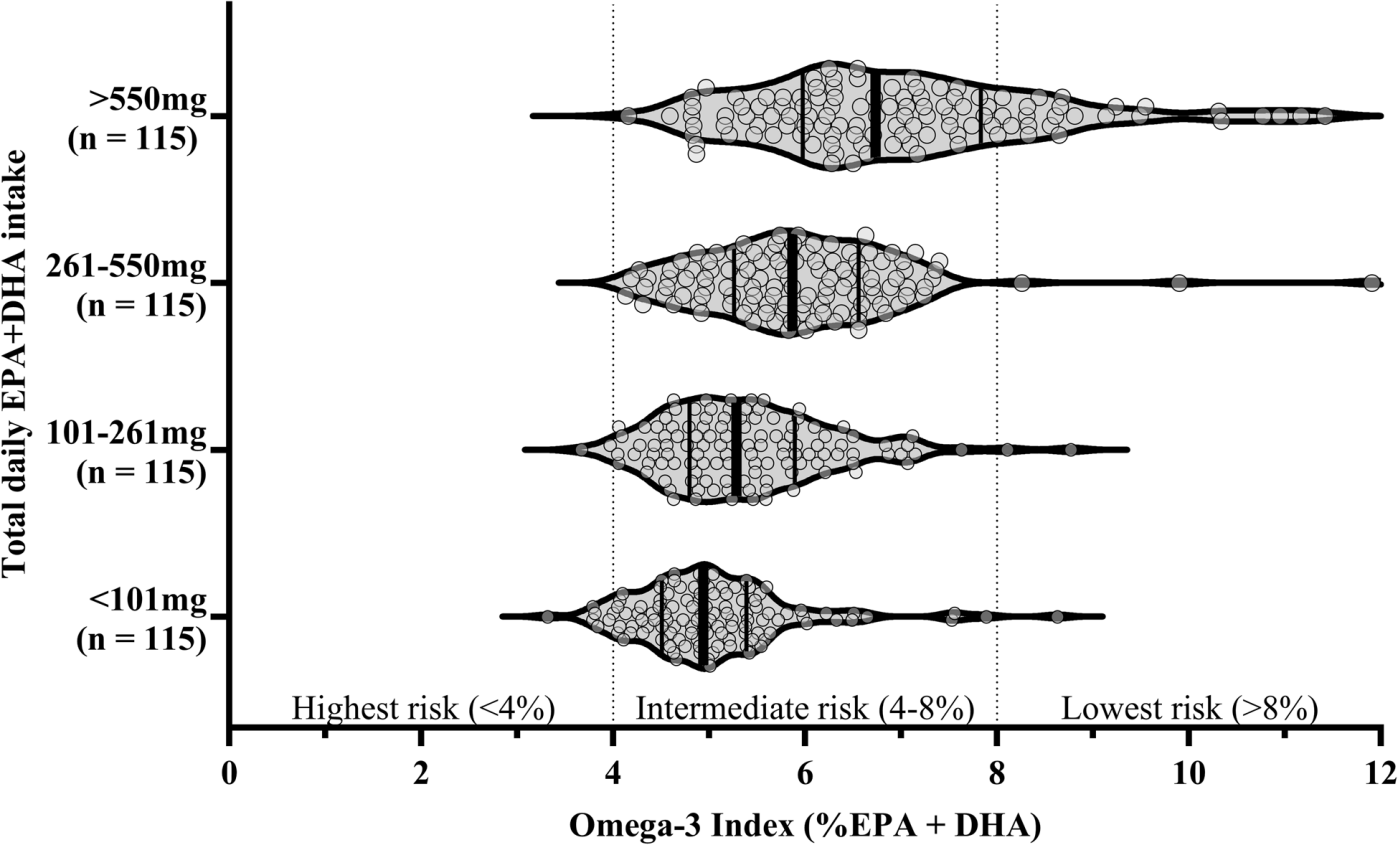
Distribution of O3I values in a cohort of highly trained, elite and world class (Tier 3–5) Australian-based male and female athletes across quartiles of total daily DHA and EPA intake. Violin plots display the distribution density, with individual observations shown as open circles. The central thick bar represents the median, and the thin bars indicate the interquartile range. Vertical dashed lines denote commonly used O3I risk thresholds (highest risk: < 4%; intermediate risk: 4–8%; lowest risk: ≥ 8%) as defined by Harris [10]

The mean (± SD) O3I of the cohort was 5.88 ± 1.40% (Fig. 1). Fifteen athletes (3%) had an O3I below 4%, 358 athletes (60%) had levels between 4–6%, 179 athletes (30%) had levels between 6–8%, and 43 athletes (7%) had levels ≥ 8%. Individual O3I values ranged from 3.32% to 12.05%. Mean O3I was significantly higher in males than females (6.06 ± 1.45% vs 5.64 ± 1.30%; independent-samples t-test, p < 0.01). O3I differed significantly across total daily DHA and EPA intake categories among athletes with available dietary data (n = 460; Kruskal–Wallis H = 131.09, p < 0.001), with progressively higher O3I values observed across increasing intake categories (Fig. 2). O3I was higher in athletes reporting omega-3 supplement use compared with non-users (7.02 [IQR: 6.28–8.01%] vs 5.38 [IQR: 4.82–6.02%]; Mann–Whitney U = 28,194.5, p < 0.001).

### DHA and EPA from Whole Food Consumption

Among athletes reporting fish consumption, the most selected portion size was 113 g (31%), followed by 170 g (28%) and 85 g (27%). Oily fish was the most frequently consumed seafood type (Table 3), with approximately one third of athletes reporting consumption at least once per week. Canned tuna was also commonly consumed, whereas other seafood types, including trout, molluscs, crustaceans, and crab, were consumed infrequently by most athletes. Across seafood categories, most athletes reported consumption less than weekly or no consumption.

**Table 3:** Self-reported frequency of seafood consumption by type

Seafood type	Never	< Weekly	Once weekly	≥ Twice weekly
Oily fish	96 (21%)	200 (44%)	109 (24%)	45 (10%)
Canned tuna	185 (41%)	134 (30%)	61 (13%)	73 (16%)
Trout	394 (88%)	50 (11%)	3 (1%)	2 (< 1%)
Whitefish	138 (30%)	274 (60%)	32 (7%)	9 (2%)
Molluscs	285 (63%)	158 (35%)	6 (1%)	1 (< 1%)
Crustaceans	211 (47%)	219 (49%)	16 (4%)	3 (1%)
Crab	393 (88%)	49 (11%)	3 (1%)	1 (< 1%)

Values are n (%)

Responses of ‘I don’t know/refuse to answer’ were treated as missing and excluded from analyses

### DHA and EPA Supplement Consumption

Eighty-nine athletes (n = 89) reported omega-3 supplement use, representing 19% of athletes who completed the dietary assessment (n = 460). Sixteen distinct supplement brands were reported, with Human and Supplement Testing Australia (HASTA)/Informed Sport certified products comprising the largest proportion (44%). A substantial proportion of athletes reported an unknown supplement brand (37%), while the remainder reported use of non-HASTA/Informed Sport certified products. Among supplement users, half reported supplementation for more than three months, with 21% reporting use for one to three months and 29% for less than one month. Supplement use frequency varied, with 29% reporting use on one to three days per week, 17% on four to six days per week, and 54% reporting daily use. Most supplement users reported consuming one to two capsules per day (80%), while 19% reported consuming three to five capsules per day and 1% reported consuming more than six capsules per day.

### Total DHA and EPA Daily Intake

Total DHA and EPA daily intake showed a wide distribution across intake categories. Values are presented as median ± interquartile range unless otherwise stated. Twenty-five percent of athletes reported intakes < 100 mg/day, 47% reported intakes of 100–500 mg/day, and 28% reported intakes > 500 mg/day. Intake of DHA, EPA and the sum of DHA + EPA from whole-food sources were generally modest (Table 4) and did not differ between males and females (189 ± 287 mg/day vs 226 ± 328 mg/day, respectively; Mann–Whitney U = 24 252.5, Z =  − 1.47, p = 0.143). Whole-food DHA and EPA daily intake was modestly higher among athletes reporting omega-3 supplement use compared with non-users (266 ± 396 mg/day vs 189 ± 299 mg/day; p = 0.031). Total DHA and EPA daily intake did not differ by sex (242 ± 432 mg/day in males vs 277 ± 487 mg/day in females; U = 24 381.5, Z =  − 1.38, p = 0.169). However, total DHA and EPA daily intake was substantially higher among supplement users (1071 ± 1148mg/day)reflecting the contribution of supplemental intake.

**Table 4:** Total self-reported DHA and EPA daily intake from whole-food and supplemental sources

Variable	Whole food intake	Supplemental intake^†^	Total intake
N	460 *(M:53%,F:47%)*	89 *(M:58%,F:42%)*	460 *(M:53%,F:47%)*
EPA + DHA (mg/day)	208 ± 87–482 (0–3430)	720 ± 321–1350 (129–3600)	261 ± 97–550 (0–5211)
EPA (mg/day)	76 ± 27–143 (0–1349)	429 ± 193–810 (77–2160)	102 ± 37–219 (0–2803)
DHA (mg/day)	127 ± 42–233 (0–2081)	270 ± 128–540 (51–1440)	158 ± 56–328 (0–2408)

Values are presented as median ± IQR (range) due to skewed distributions. ^†^Supplemental intake statistics are restricted to athletes reporting omega-3 supplement use (n = 89). Zero values represent valid zero intake

## Discussion

This study provides the first large-scale, multi-sport, real-world description of the O3I in highly trained, elite and world class (Tier 3–5) athletes, using a cohort based in Australia. Four key findings emerged. The mean O3I of the cohort was 5.88%, representing the highest reported in an athlete cohort, although only ~ 7% of individuals achieved the ≥ 8% cardiovascular disease risk reduction threshold. Self-reported DHA and EPA intake above 550 mg/day achieved on average a higher O3I, yet only the highest intake quartile for this subgroup approached or exceeded 8%. Dietary behaviours indicated low adherence to whole-food recommendations from the Australian National Heart Foundation [32], with only ~ 10% self-reporting ≥ 2 oily fish meals per week. Males exhibited higher O3I than females despite similar self-reported DHA and EPA intakes. Together, these findings provide a novel framework for interpreting O3I and its determinants in athletic cohorts.

The mean O3I of this Australian athlete cohort was 5.88%, with only ~ 7% of individuals achieving the ≥ 8% threshold associated with reduced cardiovascular disease risk. While this exceeds the 4.43% reported in a systematic review of physically trained individuals [33], it remains broadly consistent with previous athlete cohorts and only modestly higher than the < 1% of German winter endurance athletes reported to achieve an O3I ≥ 8% [11]. Despite the modest relative improvement observed, most athletes did not achieve the ≥ 8% cardiovascular disease risk reduction threshold, reflecting habitual dietary patterns insufficient in preformed DHA and EPA to support tissue incorporation, including in skeletal muscle [34, 35]. Encouragingly, only 3% of athletes in the current cohort had an O3I < 4%, compared with approximately 33% of Division I college football athletes previously reported to fall below this threshold [36]. These findings highlight the influence of habitual dietary patterns on lower-end O3I and reinforce the importance of awareness of the dietary determinants of O3I in athletic populations.

The O3I observed in this study indicates that only a small proportion of athletes achieved recommended LCn-3PUFA intakes [37]. Current Australian and New Zealand adequate intake values for total long-chain omega-3 fatty acids (EPA, DHA and DPA combined) are substantially lower, set at 90 mg/day for females and 160 mg/day for males [38], which likely contributes to O3I levels below the ≥ 8% cardiovascular disease risk threshold, while athlete-specific guidance remains limited. Consistent with population-level evidence linking DHA and EPA to long-term health [39], only 10% of athletes reported consuming ≥ 2 oily fish meals per week, while supplement use (19%) exceeded whole food intake. Low fish consumption is commonly reported in athlete cohorts [16], and is not consistently offset by supplement use, which has been reported to range from 10–22% across athletic populations [12, 16, 40]. Nevertheless, while supplement use achieved a higher O3I in some athletes, many supplement users did not achieve ≥ 8% [16, 17], consistent with previous reports, suggesting that total intake, including whole foods, as well as the duration and consistency of intake, are important determinants.

Proposed intakes for athletes range from 1000–2000 mg/day [41], with the International Olympic Committee more recently providing generalised guidance suggesting ~ 2000 mg/day of omega-3 fatty acids [42]. Despite this, only 4.5% of the cohort exceeded 2000 mg/day, a level well below intakes of up to 5000 mg/day DHA and EPA considered safe by the European Food Safety Authority [43], indicating that higher intakes are uncommon in practice. This may reflect the practical challenges of maintaining higher daily supplemental intakes. Nevertheless, intake-response data suggest an approximate 1% increase in O3I per additional 500 mg/day of DHA and EPA over time, albeit with variability between individuals [44]; similarly, in a whole-food context, the addition of one fish meal per week has been associated with an average increase of ~ 0.27% in O3I in athlete cohorts [12]. Furthermore, a daily intake of 700 mg DHA and EPA over 8 weeks has been demonstrated to elevate O3I in trained individuals by ~ 2% [45, 46], and evidence in a cohort of elite professional cyclist observed consistent daily DHA and EPA intake of ~ 1500 mg/day resulted in the most consistent O3I ≥ 8% for the athletes across a competitive cycling season, including the Tour de France [47]. Collectively this supports the importance of consistent, dietary achievable intake patterns over longer term. However, the cross-sectional design of the current study precludes causal inference, and dietary intake was assessed via self-report, which is subject to recall bias. In addition, intake data were available for a subset of participants (n = 460) relative to those who completed blood testing (n = 595).

The O3I is commonly interpreted in relation to cardiovascular disease risk, with values ≥ 8% associated with improved cardiovascular health outcomes [48] including reduced risk of sudden cardiac death [49]. Highly trained athletes develop sport-specific cardiac adaptations in response to sustained training loads [50, 51]. These adaptations vary by discipline, with endurance training associated with greater cardiac strain, including right ventricular dysfunction, while team-sport athletes experience a more heterogeneous load due to combined endurance and strength demands. Within this context, an increased risk of atrial fibrillation has been observed in athletes with a strong aerobic training base [52], highlighting the potential long-term implications of these adaptations. In this context, LCn-3PUFA, particularly DHA, play a supportive role in cardiac function [3]. Although debate exists regarding omega-3 intake and atrial fibrillation risk [53], population-level evidence does not support a detrimental effect [54], and DHA incorporation into myocardial tissue has been associated with anti-arrhythmic properties [3]. Mechanistically, DHA may improve cardiac efficiency, with evidence in trained cyclists demonstrating reduced rate-pressure product during exercise following DHA supplementation [55]. Additionally, reductions in exercising heart rate have been observed in elite Australian Rules Football (AFL) athletes [56], alongside improved heart rate recovery following high-intensity cycling [45]. In addition, higher intakes of LCn-3PUFA (> 3 g/day DHA + EPA) contribute to improved vascular function through reductions in blood pressure and circulating triglycerides [3], although such intake levels were not commonly achieved in the current cohort.

Female athletes in the current cohort exhibited lower O3I values than males, despite no clear differences in reported DHA and EPA intake. This was accompanied by higher whole blood concentrations of precursor fatty acids (LA and ALA) and lower whole blood concentrations of AA, EPA and DHA, consistent with recent observations in contact sport athletes [22]. While some studies in elite athletes have reported no sex differences [12, 57], these inconsistencies likely reflect the influence of supplement use and other behavioural factors in free-living populations. From a mechanistic perspective, females are thought to have greater enzymatic capacity for conversion of precursor fatty acids to EPA and then DHA [58], which, alongside smaller body size, may theoretically favour higher O3I levels. However, the contribution of endogenous conversion to circulating DHA is minimal, accounting for only a small proportion of sex differences [58]. Instead, direct dietary intake of DHA and EPA remains the dominant determinant of O3I [59]. Collectively, these findings suggest that sex-based differences in O3I are more strongly influenced by intake behaviours than by physiological capacity for fatty acid conversion.

Improving O3I in athletes requires multifaceted strategies, given the consistently low DHA and EPA intakes and O3I values reported across athletic populations. Sports dietitians can use behaviour change frameworks to identify athlete-specific cognitive, social and environmental barriers and enablers to dietary adherence [60]. This process can inform tailored interventions that combine education with practical strategies to increase the availability of oily fish and seafood within performance meal provision, develop food preparation skills, and support consistent use of dietary and supplemental DHA and EPA sources [61]. Whether DHA and EPA are obtained from food, supplements, or a combination of both, strategies should support regular intake at an appropriate, feasible dose. This may be particularly relevant given that 29% of supplement users in the present study reported consuming fish oil supplements only one to three days per week. Although reasons for intermittent use were not assessed, evidence from healthy and clinical adult populations suggests that daily multi-pill regimens may be undermined by tablet burden, dislike of or difficulty swallowing oral formulations, forgetfulness, uncertainty about the regimen, or beliefs that supplementation is unnecessary [62, 63]. Consistent with these barriers, a 30-day trial requiring six capsules daily found mean adherence of 62 to 78%, with complete adherence achieved by only 8 to 20% of participants across strategies [64].

Although the cross-sectional design precludes causal inference and dietary and supplement use were self-reported, the large, multi-sport cohort of highly trained athletes and concurrent assessment of O3I and intake are important strengths of this study. These findings therefore provide a robust descriptive basis for future longitudinal, intention-to-treat studies testing the multifaceted, biomarker-guided strategies outlined above, including the dose, duration and behavioural approaches required to achieve sustained improvements in O3I.

## Conclusions

Consistent with historical observations in physically trained cohorts, the current findings demonstrate that O3I values ≤ 6% remain prevalent in the context of Tier 3–5 male and female athletes, with relatively few individuals achieving the ≥ 8% cardiovascular disease risk threshold. This represents a missed opportunity to support cardiovascular function during competition and promote life-time nutritional health behaviours. In this context, the O3I represents a modifiable marker associated with cardiovascular health and provides a practical, behaviourally driven approach to supporting cardiac and vascular function across the athlete lifespan.

## Data Availability

All data relevant to the study are included in the article. Anonymised data available upon reasonable request.
